# A Simple Laser Ablation-Assisted Method for Fabrication of Superhydrophobic SERS Substrate on Teflon Film

**DOI:** 10.1186/s11671-018-2658-3

**Published:** 2018-08-22

**Authors:** Fangjia Chu, Sheng Yan, Jiangen Zheng, Lingjun Zhang, Haiyan Zhang, Keke Yu, Xiaonan Sun, Anping Liu, Yingzhou Huang

**Affiliations:** 1 Chongqing Key Laboratory of Soft Condensed Matter Physics and Smart Materials, College of Physics, Chongqing University, Chongqing, 400044 China; 2 Department of Applied Physics, College of Physics, Chongqing University, Chongqing, 400044 China; 3 Department of Physics, The Hong Kong University of Science and Technology, Clear Water Bay, Kowloon, Hong Kong, China

**Keywords:** Surface-enhanced Raman scattering, Teflon, Laser-treatment, Superhydrophobic

## Abstract

With high sensitivity at single molecule level, surface-enhanced Raman scattering (SERS) is considered as an ultrasensitive optical detection technology with broad application prospects in lots of fields. However, the complicated fabrication and unaffordable price of SERS substrate are still a roadblock on the way to be widely used in industry. In this work, the SERS spectra on a commercial laser engraved Teflon (PTFE) film with engraved microarray are investigated. The wettability of film surface modulated by laser engraving make the microarray have the ability to decrease the contact area on film surface while water evaporation. The SEM image of the engraved area points out the micro/nanostructures generated engraving process is crucial to its superhydrophobic property. The probing molecules (i.e., methylene blue and rhodamine6G) were utilized to investigate with the limit of detection (1 × 10^−14^ M). Furthermore, the biomolecule (bovine serum albumin) was used to demonstrate its benefits in biological applications. The measured intensities of Raman spectra on this PTFE with laser engraved microarray demonstrate its potential value for a SERS substrate. Our work on this simple, cheap SERS substrate with high sensitivity has a great commercial value and plenty of application in lots of fields.

**Electronic supplementary material:**

The online version of this article (10.1186/s11671-018-2658-3) contains supplementary material, which is available to authorized users.

## Background

Surface-enhanced Raman scattering (SERS) has already aroused great interests among the public since its discovery in 1974, because it is a promising ultrasensitive spectroscopic technique to obtain the vibrational fingerprint of characteristic molecular even in the case of super-dilute solution [1–5]. The huge electromagnetic field near metal surface is the dominated enhanced factor in SERS, which come from the light excited collective oscillation of free electrons called surface plasmon. As a result, the obtained strong molecular Raman signals are mainly derived from those molecules which are located in the nano-gaps or clefts, so-called hot-spots, near metal surface where the electromagnetic field is greatly enhanced.

In the previous works, the various morphology Ag or Au nanoparticles were introduced to deposit on the glass or silicon wafer in order to fabricate the SERS substrate [6–9]. Unfortunately, the glass, silicon wafer, and other frequently used substrates are hydrophilic, so the nanoparticles dispersed in solvent are freely dispersed on the substrates after the evaporation, resulting the distance between the nanoparticles so large that it is hard to form a larger electromagnetic field enhancement. Considering the diffusion of solute, there is a method that can be expected to succeed in concentrating the solutes in a small area, obliging the nanoparticles to be densely packed together and molecules to get into the hot-spot areas, which could achieve the goals enhancing the Raman signals of molecules. Hence, the train of thought provides another approach to fabricate the impactful SERS substrates. Recently, based on the conception, various hydrophobic or superhydrophobic substrates have already been reported as active SERS substrates thanks to the high enhancement and improved reproducibility such as the Ag-NP decorated Si cylindrical micro-pillar array substrate, silver nanoparticles coated zinc oxide nanorods array superhydrophobic substrate, and so on [10, 11]. The reason is that the superhydrophobic surface could effectively assemble the solutes dissolved in the solution into a small range after the water evaporation. However, many superhydrophobic substrates cause the losing of solutes because of their inherent micro/nanostructures [12, 13]. Meanwhile, the process of fabricating those substrates is often time-consuming and complex, and those substrates usually are expensive. Normally, it takes several hours for the whole water volatilizing at room temperature, limiting the rapid detection and analysis in practical applications. On account of those drawbacks, it is a challenge to popularize the superhydrophobic SERS substrate widely in the real world.

In this paper, a laser ablation-assisted method was proposed to fabricate the SERS substrate on the Teflon (PTFE). The wettability of the surface is changed by the laser engraving technology. By designing a suitable laser engraving pattern and setting the appropriate parameters of engraving, one kind of superhydrophobic PTFE substrate with microarrays is obtained. The microarrays are hydrophobic and surrounded by the superhydrophobic area, which is generated from the laser engraving.

Thanks to the special substrate, the solutes dissolved in the water can be successfully collected in the hydrophobic small circles after the solvent evaporation, taking only 10 min. Similar to the 24-well plates in biological laboratory, the developed SERS substrate with virtual wells can conveniently detect the molecules and their concentrations. Furthermore, the obtained SERS substrate costs only 20 RMB and the whole manufacturing process takes 20 min. All in all, a low-cost, reliable, practical, and active SERS substrate is fabricated, which can accomplish fast evaporation without affecting the detection results in this work.

## Methods and Experiment

### Material

Silver nitrate (99.99%), PVP (Mw = 58,000, K29-32), sodium borohydride (NaHB_4_), ethylene glycol (EG), methylene blue (MB), and Rhodamine6G (R6G) were purchased from Shanghai Aladdin biochemical Polytron Technologies Inc. (Shanghai, China). The bovine serum albumin (BSA) was purchased from Sigma-Aldrich (Taufkirchen, Germany). All chemicals were used as received without further purification or treatment. High-purity deionized water (18.25 MΩ·cm) was produced using Aquapro AWL-0502-H (Aquapro International Company LLC., Dover, DE, USA). As-purchased Teflon (PTFE) was directly used, which is widely commercially available online, and the size was 50 × 30 × 5 mm.

### The Synthesis of Ag Nanoparticles

The Ag nanoparticles were synthesized according to a previous synthetic method. In the experiment process, the EG solution was introduced to dissolve the solid or powder. In the beginning, 6 mL of an EG solution was added into a 100 mL flask and then the flask was moved into an oil bath at a temperature of 165 °C under stirring for 1 h. Next, 0.08 mL of a NaHB_4_ solution (0.0015 mg/mL), 1.5 mL of a PVP solution (20 mg/mL), and 0.4 mL of an AgNO_3_ solution (48 mg/mL) were respectively added to the previous flask in turn by stirring for 20 min. Afterward, the gray silver colloid was obtained. The Ag nanoparticles could be gained from the solution by centrifugation and washed with ethanol more than four times. In the end, the sample was dispersed in the water for further experimentation. Using the deionized water, the as-prepared Ag nanoparticles would be prepared into different concentrations of silver colloid and the concentrations were estimated to be 1.19 × 10^−11^, 1.19 × 10^−12^, 1.19 × 10^−13^, 1.19 × 10^−14^, and 1.19 × 10^−15^ M.

### The Fabrication of Engraved PTFE

The purchased original PTFE was rinsed with the water and ethanol for more than three times. Then, the washed original PTFE was engraved by the CO_2_ laser engraving machine on the basis of the CAD design shown in Additional file 1: Figure S1 using laser engraving (the output power: 16–24%, the engraving speed: 35–75 mm/s, the engraving step length: 0.02–0.10 mm).

### Characterization

The surface morphology of engraved and original PTFE was obtained by the SEM (TESCAN MIRA 3 FE). Five microliters of Ag nanoparticles aqueous solution was dropped on the original PTFE and engraved PTFE respectively, and then the evaporation process in the room temperature and the images of static water contact angle were obtained using high-speed camera (Phantom V 7.3). The value of static water contact angle was measured by one type of ruler commercial software.

Two drops of 5 μL Ag solution were dropped on the original PTFE and the engraved PTFE respectively. Subsequently, those would be put into an oven (70 °C). After the evaporation, the Ag nanoparticles aggregated on the two surfaces were characterized by the optical microscope and the SEM, respectively. Another drop of 5 μL Ag colloid aqueous solution was dropped on the engraved PTFE, and the SEM images of Ag nanoparticles aggregates were obtained after the room temperature evaporation.

In a typical SERS analysis, Ag aqueous solution with the same concentration and volume (5 μL) was deposited on the original PTFE substrate and the engraved PTFE substrate respectively to form an enhanced basement. Then, 5 μL of aqueous solution of the MB and R6G with different molar concentrations (10^−9^, 10^−11^, 10^−12^, 10^−13^, and 10^−14^ M) was placed on the enhanced substrate as a probe and dried in the drying oven (70 °C), and the SERS activity was measured with the Raman spectrograph with a 633 nm He–Ne laser (10 mW). As reported, the molecules could keep the SERS activity at this temperature [12]. Five microliters of aqueous solution of the BSA with different concentrations (20, 2, 0.2, 0.02 and 0.002 μg/mL) was placed on the enhanced substrate and dried in the oven (40 °C), and the SERS activity was measured with the Raman spectrograph with a 633 nm He–Ne laser (10 mW). To keep the bioactivity of BSA, the evaporation temperature was set up to 40 °C [14]. The signals were obtained with one scan every 20 s in all measurements.

## Results and Discussion

**Fig. 1:**
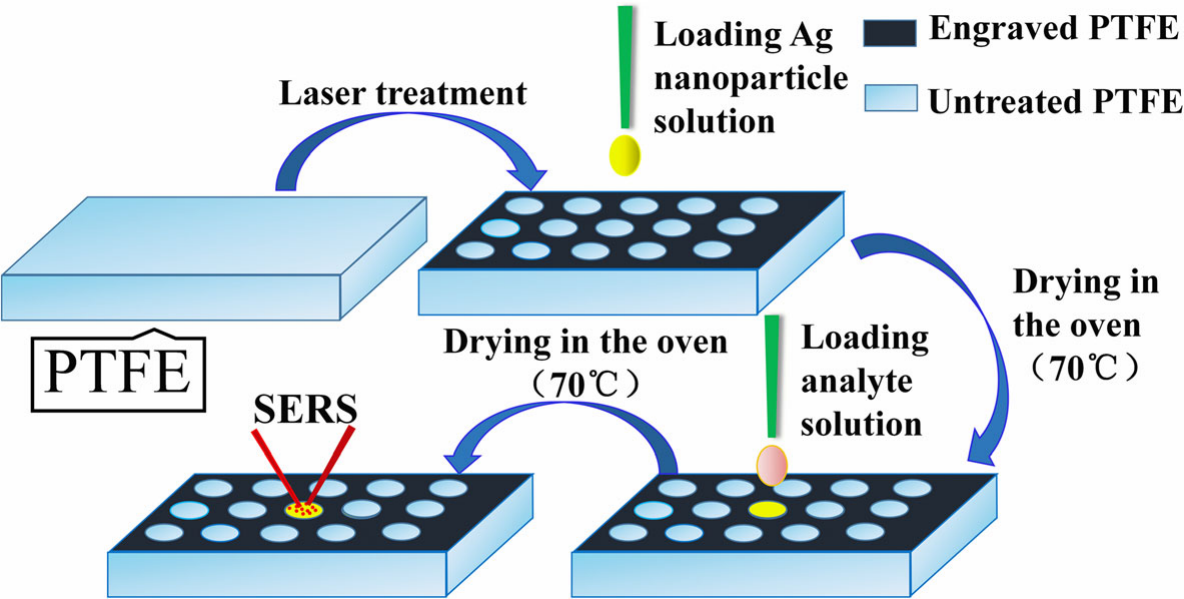
The schematic diagram of experimental process

The process of experiment is shown in Fig. 1. The wettability of the original PTFE surface was changed by laser treatment using the CAD design shown in Additional file 1: Figure S1, leading to a result that the whole surface became superhydrophobic except for these untreated areas, which were the hydrophobic surface (the diameter of circle: 0.5 mm, gap: 0.8 mm). The photograph of engraved PTFE is shown in Additional file 1: Figure S2. Afterward, a drop of Ag colloidal solution (5 μL) was dropped on the engraved PTFE and evaporated in the oven (70 °C). About 10 min later, the Ag nanoparticles could be aggregated into the circle (hydrophobic surface) due to the highly repulsive property of superhydrophobic surface, and then the active SERS substrate was obtained. Firstly, the Rhodamine6G (R6G) and methylene blue (MB) were acted as the probe molecules to investigate the SERS performance of the fabricated SERS substrate. One drop of molecular solution was dropped on the engraved PTFE covering the previously deposited Ag nanoparticles. Because of the water repellency of superhydrophobic structure, the molecular droplet would become thicker and thicker in the process of evaporation, which would enrich the molecules to the hot-spot areas at the gap between nanoparticles effectively. Interestingly, the evaporation of high temperature could not only accelerate the solvent evaporation to achieve rapid analysis without influencing the experiment results, but also hardly pay an adverse effect on the aggregation of solutes at 70 °C.

**Fig. 2:**
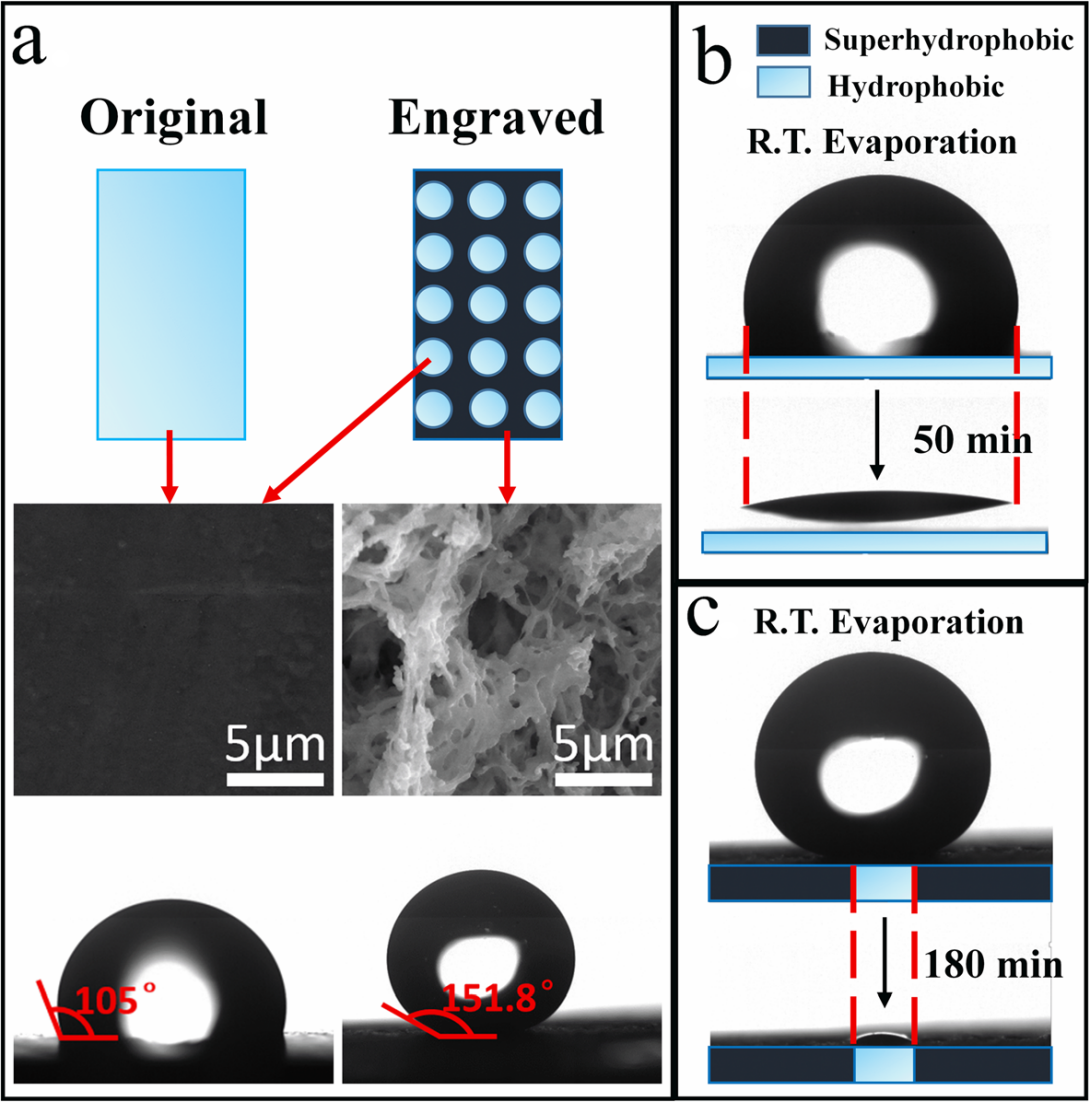
**a** The SEM images of substrate surface about original PTFE and engraved PTFE and corresponding static contact angle images; **b** Evaporation profiles of solution on the original PTFE; **c** Evaporation profiles of solution on the engraved PTFE

To further explain the reason that the engraved PTFE is better than original PTFE on the ability to enrich solutes, the SEM images and the evaporation profiles using a high-speed video camera of the two types PTFE substrates are obtained and shown in Fig. 2. As the PTFE was engraved, the laser would destroy and ablate the smooth surface of original PTFE, which could change the roughness of the surface and let the micro/nanostructures appear on the PTFE. In Fig. 2a, for engraved PTFE, all untreated circles’ surfaces show a relatively smooth surface, but the engraved area is embellished with micro/nanostructures, which turns the PTFE into superhydrophobic PTFE. Meanwhile, the contact angle images show that the static water contact angle of engraved PTFE is much bigger than the original one and the value of angle (engraved PTFE) is 151.8° as shown in the bottom of Fig. 2a, which has already reached the value of the static contact angle required by the superhydrophobic structure [15]. The high-speed video camera was used to observe and record the evaporation process that a drop of 5 μL Ag colloid solution evaporated on the original and engraved PTFE at room temperature (R.T.), respectively. Because the evaporation processes took a long time, the evaporation profiles of the start and the end of the evaporation process were captured respectively to explain intuitively the process, shown in Fig. 2b (original PTFE) and Fig. 2c (engraved PTFE). For the original PTFE, during evaporation, the contact surface between the solution and the substrate surface barely decreases. By contrast, there exists a relatively obvious decrease about the contact surface for engraved PTFE as shown in Fig. 2c. The reason is that the water repellency of the micro/nanostructures gradually shrinks the droplet into the hydrophobic circle area during the R.T. evaporation, contributing to the decrease of contact area. By comparing Fig. 2b and c, it could be observed directly that the Ag nanoparticles on the fabricated PTFE were collected in a much smaller area than original PTFE. It is important to highlight that the special superhydrophobic surface (the alternate distribution of hydrophobic and superhydrophobic surface) does not let the solution pin to the micro- or nanoscale textures and the majority of solutes would be collected in these circles after the evaporation as shown in Additional file 1: Figure S3. Put it another way, the special superhydrophobic surface can avoid disadvantages of general superhydrophobic materials that the analytes remain in the micro/nanostructures after the evaporation, causing the loss of solutes and a weakening of SERS signals.

**Fig. 3:**
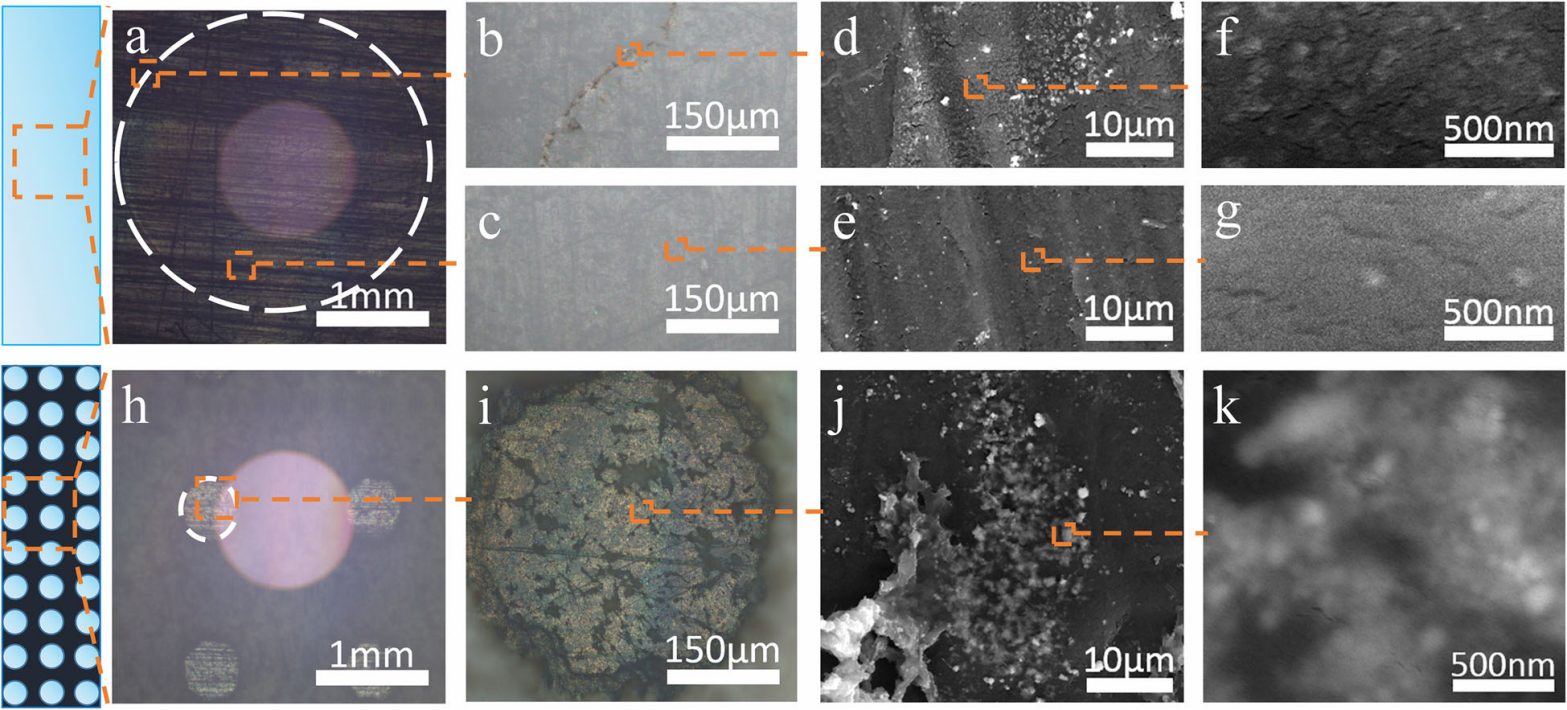
**a**–**c** The optical microscope images and **d**–**g** the SEM images of Ag nanoparticles aggregation on the original PTFE with different multiples. **h**, **i** The optical microscope images and **j**, **k** the SEM images of Ag nanoparticles aggregation on the engraved PTFE with different multiples. The whole orange small boxes represent the magnified area and the white dotted line circles the finial area of Ag nanoparticles accumulation

In order to visually observe that the Ag nanoparticles have more densely gathered together on the engraved PTFE compared with the original PTFE under high-temperature evaporation (70 °C), there are optical microscope images and SEM images with different multiples as shown in Fig. 3, respectively. The SEM image of as-prepared Ag nanoparticles is shown in Additional file 1: Figure S4 [16]. Due to the coffee ring effect, after all the water completely evaporated, a majority of Ag nanoparticles would gather in the edge and the remaining nanoparticles would be dispersed in the middle which occupies the most of area for the original PTFE as shown in Fig. 3a–g. With regard to engraved PTFE, after the evaporation process in a high-temperature environment, the Ag nanoparticles would accumulate in the little circle and there was no coffee ring effect, as shown in Fig. 3h–k. It should be pointed out that the final area of Ag nanoparticles aggregation on the engraved PTFE is almost 25 times smaller than the original PTFE by comparing Fig. 3a and h. To reduce the time of evaporation, the sample was put into an oven. Notably, the high-temperature evaporation could make nanoparticles more compact compared to the R.T. evaporation as shown in Additional file 1: Figure S5. A possible explanation is that rapid evaporation could make the Ag nanoparticles mass together more quickly. However, the evaporation temperature cannot be increased too high because the structure of analyte molecules might be deteriorated at very high heating temperatures, resulting in the reduced SERS signals. Because of the rapid evaporation, it could save much time to prepare the SERS substrate. To sum up, the fabricated substrate can efficiently enrich the solutes into a much smaller area within 10 min for 5 μL Ag solution.

Since sensing ability of engraving PTFE highly depends on the hydrophobicity of surface, the size of sensing area, and the initial concentration of Ag nanoparticles, we investigate these parameters by fabricating the designated PTFE substrate. The main Raman peak intensity at 1322 cm^−1^ of MB (1 × 10^−9^ M) is obtained on the various SERS substrates. We investigate the effect of the engraved step length, the output power, the engraving speed, and the size of circle’s diameter on the hydrophobicity of surface. It should be pointed out that the engraved step length, the output power, the engraving speed, and the circle’s diameter would restrict each other and influence the sensing ability of substrate. To understand more clearly how each factor affects the sensing ability, the three variables remain the same and one of them is changed.

**Fig. 4:**
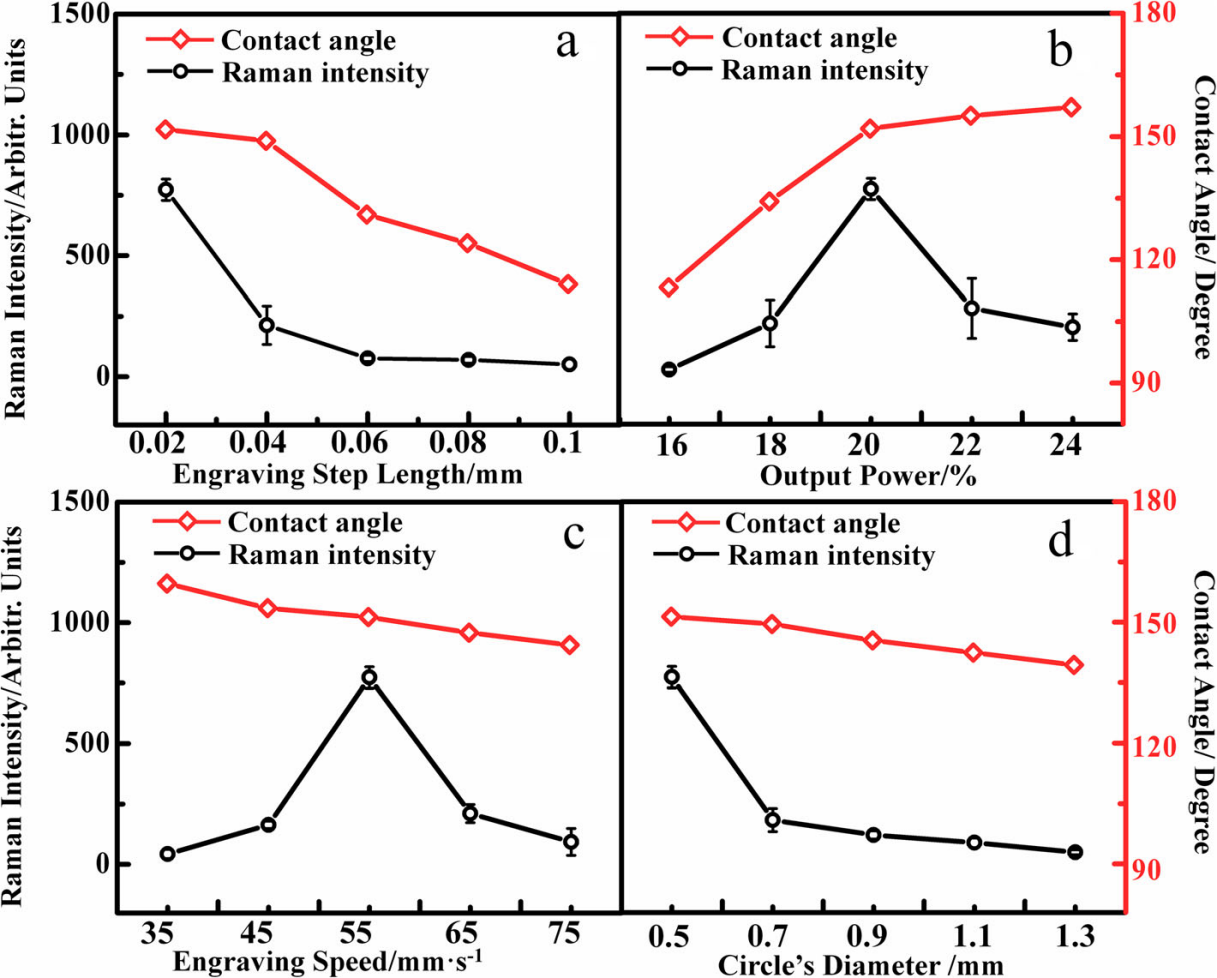
**a** The relation between the contact angle, Raman intensity, and the engraving speed (the output power: 20%; the engraving speed: 55 mm/s; the circle’s diameter: 0.5 mm; the Ag concentration: 1.19 × 10^−12^ M). **b** The relation between the contact angle, Raman intensity, and the output power (the engraving step length: 0.02 mm; the engraving speed: 55 mm/s; the circle’s diameter: 0.5 mm; the Ag concentration: 1.19 × 10^−12^ M). **c** The relation between the contact angle, Raman intensity, and the engraving speed (the engraving step length: 0.02 mm; the output power: 20%; the circle’s diameter: 0.5 mm; the Ag concentration: 1.19 × 10^−12^ M) **d** The relation between the contact angle, Raman intensity, and the circle’s diameter (the engraving step length: 0.02 mm; the output power: 20%; the engraving speed: 55 mm/s; the Ag concentration: 1.19 × 10^−12^ M)

As is shown in Fig. 4a, the contact angle (red line) and the Raman intensity (black line) decrease with the increase of the engraving step length. The reason is that the smaller the engraving step length, the denser the micro/nanostructures. With the aid of the denser micro/nanostructures outside the circles, the solutes could be successfully enriched into these small circles, and then the sensing ability of the substrate is improved. Due to the limitation of the laser engraving machine precision, 0.02 mm is the minimum engraving step length in this work. As is shown in Fig. 4b, with the increase of the output power, the contact angle increases but the Raman intensity shows a trend of first increase and then decrease. With the increase of the output power, the original PTFE was destroyed and ablated by the strong laser, leading to more micro/nanostructures on the surface of the substrate. Due to the more micro/nanostructures, the surface of the substrate becomes more hydrophobic, as evidenced by the increase of contact angle. Notably, the superfluous micro- or nanostructure had an adverse effect on the enhancement of the molecular Raman signals. The reason is that the sufficient micro/nanostructures make the substrate superhydrophobic, which is able to enrich the analytes into the hydrophobic circle, but the superfluous PTFE fragments are easy to cover the small hydrophobic circles as the increase of the laser power. Subsequently, the solutes remain on the micro/nanostructures after evaporation process causing the loss of solutes, which leads to a weakening of the Raman intensity. It could be concluded that the engraved PTFE fabricated by the 20% output laser is the optimal SERS substrate.

In Fig. 4c, the contact angle decreases and the Raman intensity climbs up and then declines as the engraving speed increases. Compared with the contact angle in Fig. 4b and Fig. 4c, it is concluded that the effect of engraving speed on the surface of PTFE is opposite to that of output power. The reason is that with the laser speed increasing, the exposure time of laser point on the PTFE surface becomes shorter, causing less original PTFE ablated. Therefore, fewer micro/nanostructures are produced, leading to the decrease of the contact angle. According to the relation between Raman intensity and the engraving speed, the SERS substrate produced by the laser speed of 55 mm/s possesses the best sensing ability. So, 55 mm/s was chosen as the engraved speed in the experiment. As is shown in Fig. 4d, the contact angle and the Raman intensity would decline with the circle’s diameter increasing. As the circle is the untreated PTFE, these areas keep its original wetting property, a hydrophobic state. When a drop of solution is dripped into the engraved PTFE substrate, the droplet tends to stay in the hydrophobic circle. Due to the repellency of water on the superhydrophobic structure beside the circle, the droplet on the engraved PTFE has a fairly large contact angle. With the increase of the circle’s diameter, the contact area between the droplet and the surface increases and the droplet would slowly go flat rather than a spheroid. Because the volume of droplet on the different substrates is the same, the contact angle decreases gradually. The effect changes the surface of engraving PTFE from superhydrophobic to hydrophobic, which could affect the enrichment of the solutes, lead to the loss of solutes and finally weaken the Raman signals. At the same time, with the circle’s diameter increasing, the Ag nanoparticles would disperse into a larger region, which would increase the gap between Ag nanoparticles and then weaken the SERS signals. On the other hand, due to the increasing of circle’s diameter, the analytes are dispersed on a larger area, making the SERS detection difficult. To sum up, the Raman intensity of the molecule would weaken with the circle’s diameter increasing. Because of the limitation of laser engraving machine’s precision, 0.5 mm is the minimal circle’s diameter.

In the meantime, the initial concentration of Ag nanoparticles also affects the SERS intensity shown in Additional file 1: Figure S6. With the increasing of the concentration of Ag nanoparticles, the Raman intensity dramatically rises and then tends to stabilize. With the increase of Ag nanoparticles, there are more “hot spots” on the substrate, leading to the increase of Raman signals. The more detailed discussion was provided in the supporting information. To save the Ag nanoparticles, the 1.19 × 10^−12^ M Ag colloid solution is chosen as the initial concentration of Ag nanoparticles. To sum up, in this work, the 0.02 mm engraving step length, the 20% output power, the 55 mm/s engraving speed, the 0.5 mm circle’s diameter, and the 1.19 × 10^−12^ M Ag nanoparticles were chosen.

**Fig. 5:**
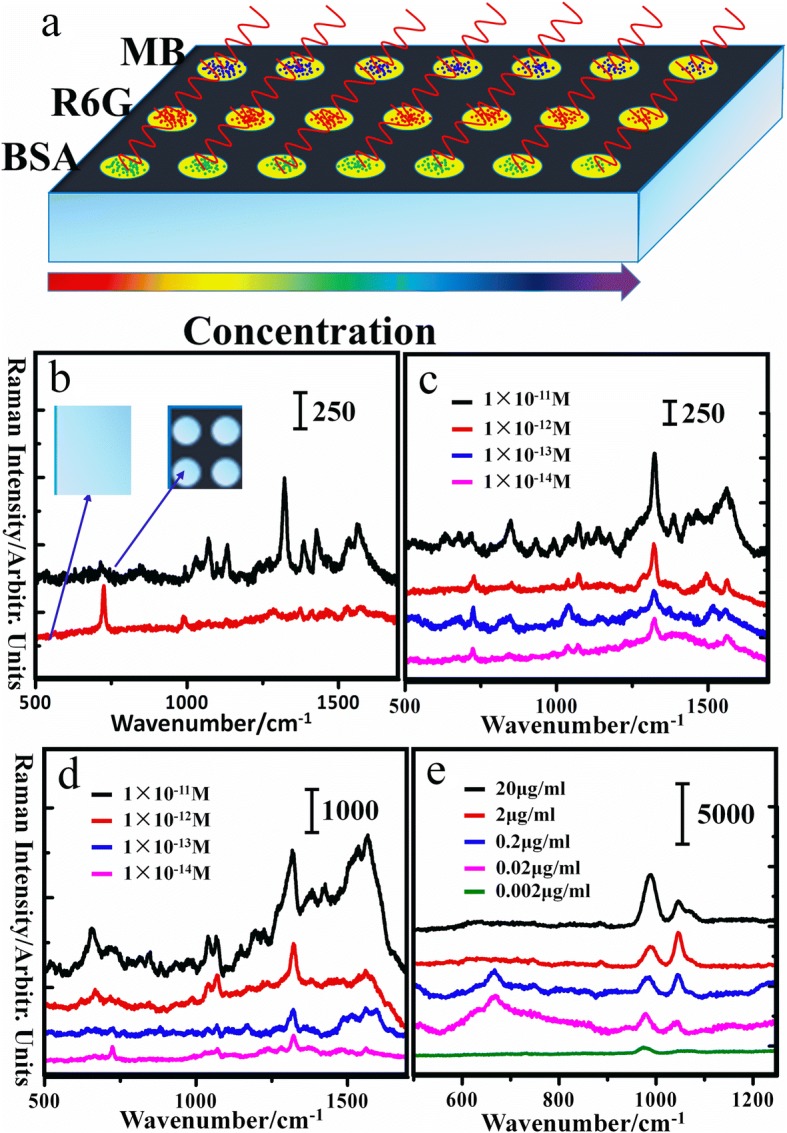
**a** The diagrammatic sketch of detecting substance on the engraved PTFE. **b** SERS spectra of MB molecules with the same concentration (1 × 10^−9^ M) was obtained on original PTFE and engraved PTFE respectively. **c**, **d**, and **e** SERS spectra of MB molecules, R6G molecules, and BSA with various concentration respectively

In order to realize multiple detections on the same substrate, the engraved PTFE was made into the 24-well plates (Fig. 5a), which were similar to the 24-well plates for cell culture. The obtained substrate could achieve detect different substance simultaneously on the same engraved PTFE. Meanwhile, the micro/nanostructures on the engraved PTFE surface could be acted as the virtual wells between two different droplets, which could prevent the different droplets from merging. To further explain the advantages of the engraved PTFE substrate with microarrays, the original PTFE was chosen for comparison. The SERS spectra of MB molecules is shown in Fig. 5b. The SERS signal intensity of MB obtained on the microarrays is an obvious huge enhancement compared with the original PTFE. It has been already known that Ag nanoparticles evaporating on the original substrate tend to diffuse over a much larger area than that on the engraved PTFE according to Fig. 3. Therefore, the Ag nanoparticles, which are dispersed on the original PTFE, are far from each other even on the edge, contributing the poor Raman signals. However, in terms of the PTFE with microarrays, it could make massive nanoparticles gather together and the reduced distance between nanoparticles can enhance SERS signals. As previously reported [17–19], the smaller the size of the gap is, the stronger the electromagnetic field of the gap between the two nanoparticles is. On the other hand, the engraved PTFE has the ability to concentrate the analyte molecules in super-diluted aqueous solutions while original PTFE does not, causing more molecules inside the focus area of the incident laser spot on the engraved PTFE compared with the original PTFE. Meanwhile, due to the superhydrophobic condensation effect, the substrate could help molecules be delivered into the hot-spot areas [20]. It is noteworthy that the high probability of acquiring SERS signals of molecules is another very important factor of active SERS substrates. To prove that the probability of detecting the MB molecules on the engraved PTFE is higher than that on the original PTFE, the systematic mapping measurements are performed and the concentration of molecules is 1 × 10^−9^ M, as shown in Additional file 1: Figure S7. Fig. 5c, d shows the spectra of MB molecules and R6G molecules respectively, which are collected on the engraved PTFE SERS substrates. It is illustrated that the Raman signals of MB molecules are gradually weakened with the decrease of molecular concentration while the main peaks could be distinguished and the limit concentration of detection is 1 × 10^−14^ M, as shown in Fig. 5c. Besides, the analogous consequence is found by the R6G spectra as shown in Fig. 5d. To prove the utilization of the engraved PTFE in biological applications, a protein, bovine serum albumin (BSA), was used to test the performance of the developed SERS substrate. The BSA with various concentrations was detected in the water, and the Raman spectra were shown in Fig. 5e. Furthermore, the limit detection of MB, R6G, and BSA detected on various substrates or using different methods is listed in the Additional file 1: Table S1.

## Conclusion

In summary, a low-cost, active, and superhydrophobic SERS substrate was fabricated by engraving the PTFE via the proper engraving parameters and pattern, which could achieve the multiple detections on one and the same substrate. By comparing the contact angle images and evaporation profiles of the original and engraved PTFE, the engraved PTFE possesses the better hydrophobicity and succeeds in decreasing the contact area on the substrate surface. Further, the SEM image of the engraved area reveals the reason that engraved PTFE possesses the better hydrophobicity due to the micro- or micro/nanostructures. What is more, the PTFE with microarrays could contribute to collect the Ag nanoparticles into a very small area compared to the original PTFE by the obtained SEM images about aggregation of Ag nanoparticles on the two substrates, leading to produce a large number of hot-spots on the engraved PTFE surface. The intensity of MB Raman spectra (10^−9^ M) obtained on the engraved PTFE is a huge enhancement compared with the original PTFE. It should be pointed out the lowest concentration of R6G and MB is 1 × 10^−14^ M detected on the fabricated superhydrophobic SERS substrate. Meanwhile, it is proved that the substrate could be used to detect the BSA (0.002 μg/mL). All in all, in this paper, a kind of cheap, highly sensitive and active SERS substrate possesses a big commercial value and can be used in lots of fields.

## Additional file

Additional file 1: **Figure S1.** The CAD image of engraving the PTFE. **Figure S2.** The photograph of engraved PTFE. **Figure S3.** a. and b. are schematic illustration of solution evaporation on the original superhydrophobic surface and the engraved PTFE surface respectively. **Figure S4.** The SEM image of as-prepared Ag nanoparticles. **Figure S5.** The SEM images of Ag nanoparticles aggregation on the engraved PTFE at the different evaporating temperatures with different multiples. a and b the evaporating temperature is 20 °C; c and d the evaporating temperature is 70 °C. The whole red small boxes represent the magnified area. **Figure S6.** The relation between the Ag concentration and the Raman intensity. **Figure S7.** SERS mapping results of methylene blue at 1322 cm^−1^ obtained from 1 × 10^−9^ M aqueous solutions on the original PTFE b and the engraved PTFE d. The measurement areas are circled by the homologous red square frame in the a and c. **Table S1.** The limit detection of MB, R6G, and BSA detected on various substrates or used different methods. (DOCX 2605 kb)

## Abbreviations

BSA: Bovine serum albumin
EG: Ethylene glycol
MB: Methylene blue
NaHB_4_: Sodium borohydride
PTFE: Teflon
R.T.: Room temperature
R6G: Rhodamine6G
SEM: Scanning electron microscope
SERS: Surface-enhanced Raman spectrum
