# Supplementary material for: A Simple Laser Ablation-Assisted Method for Fabrication of Superhydrophobic SERS Substrate on Teflon Film
Source: Nanoscale Res Lett. 2018 Aug 22;13:244. doi: 10.1186/s11671-018-2658-3 (PMC6104470; doi:10.1186/s11671-018-2658-3)
Supplement: Supplementary file 1 — Figure S1. The CAD image of engraving the PTFE. Figure S2. The photograph of engraved PTFE. Figure S3. a. and b. are schematic illustration of solution evaporation on the original superhydrophobic surface and the engraved PTFE surface respectively. Figure S4. The SEM image of as-prepared Ag nanoparticles. Figure S5. The SEM images of Ag nanoparticles aggregation on the engraved PTFE at the different evaporating temperatures with different multiples. a and b the evaporating temperature is 20 °C; c and d the evaporating temperature is 70 °C. The whole red small boxes represent the magnified area. Figure S6. The relation between the Ag concentration and the Raman intensity. Figure S7. SERS mapping results of methylene blue at 1322 cm−1 obtained from 1 × 10^−9^ M aqueous solutions on the original PTFE b and the engraved PTFE d. The measurement areas are circled by the homologous red square frame in the a and c. Table S1. The limit detection of MB, R6G, and BSA detected on various substrates or used different methods. (DOCX 2605 kb) [file 11671_2018_2658_MOESM1_ESM.docx]

**Supporting Information**

**for**

**A simple laser ablation-assisted method for fabrication of superhydrophobic SERS substrate on** **Teflon film**

Fangjia Chu[^‡^](http://pubs.rsc.org/en/content/articlehtml/2018/lc/c8lc00047f#fn2)^1,2^, Sheng Yan[^‡^](http://pubs.rsc.org/en/content/articlehtml/2018/lc/c8lc00047f#fn2)^3^, Jiangen Zheng^1^, Lingjun Zhang^1^, Haiyan Zhang^1^, Keke Yu^1,2^, Xiaonan Sun^2^, Anping Liu^*2^, Yingzhou Huang^*1^

*^1^ Chongqing Key Laboratory of Soft Condensed Matter Physics and Smart Materials, College of Physics, Chongqing University, Chongqing, 400044, China* (F. C.: chufangjia@cqu.edu.cn; J. Z.: zhengjiangen@cqu.edu.cn; L. Z.: zhanglingjun@cqu.edu.cn; H.Z.: zhanghaiyan@cqu.edu.cn; K.Y.: yukeke@cqu.edu.cn; Y.H.: yzhuang@cqu.edu.cn)

*^2^ Department of Applied Physics, College of Physics, Chongqing University, Chongqing, 400044, China* (X.S.: xnsun168@cqu.edu.cn; A.L.: liuanping@cqu.edu.cn)

*^3^ Department of Physics, The Hong Kong University of Science and Technology, Clear Water Bay, Kowloon, Hong Kong, China* (S.Y.: sy034@uowmail.edu.au)

*Corresponding authors: Anping Liu (liuanping@cqu.edu.cn); Yingzhou Huang (yzhuang@cqu.edu.cn)

[^‡^](http://pubs.rsc.org/en/content/articlehtml/2018/lc/c8lc00047f#fn2)^These authors contributed equally.^


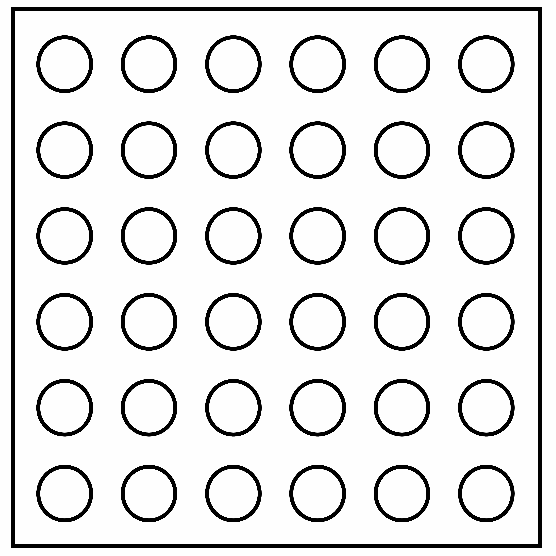


**Figure S1.**The CAD image of engraving the PTFE. The diameter of circle is 0.5mm and the distance separation of two circles is 0.8mm.


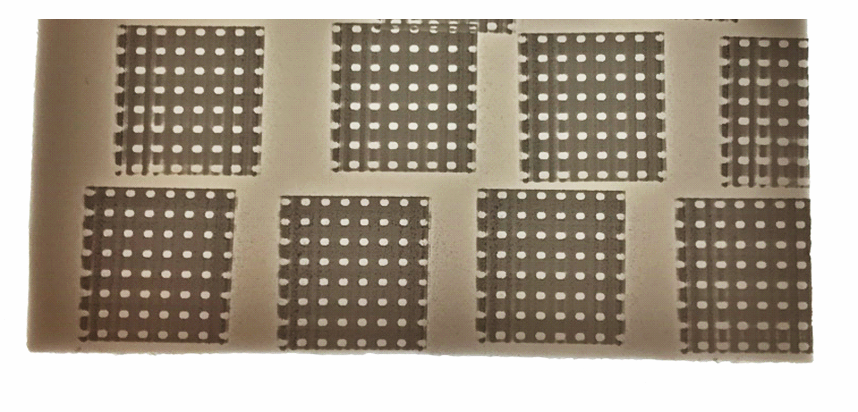


**Figure S2.**The photograph of engraved PTFE.


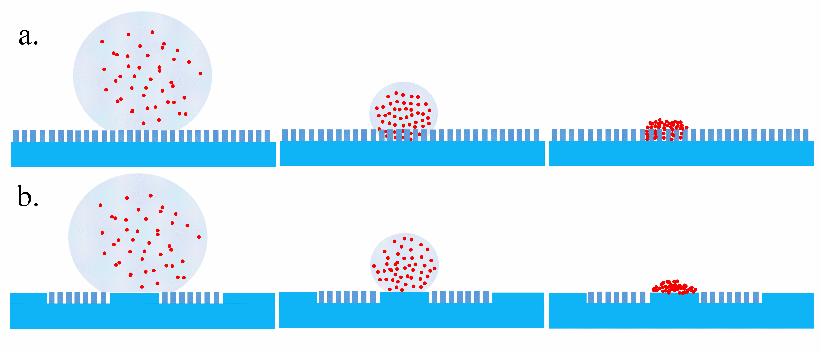


**Figure S3.** (a) and (b) are schematic illustration of solution evaporation on the original superhydrophobic surface and the engraved PTFE surface respectively.


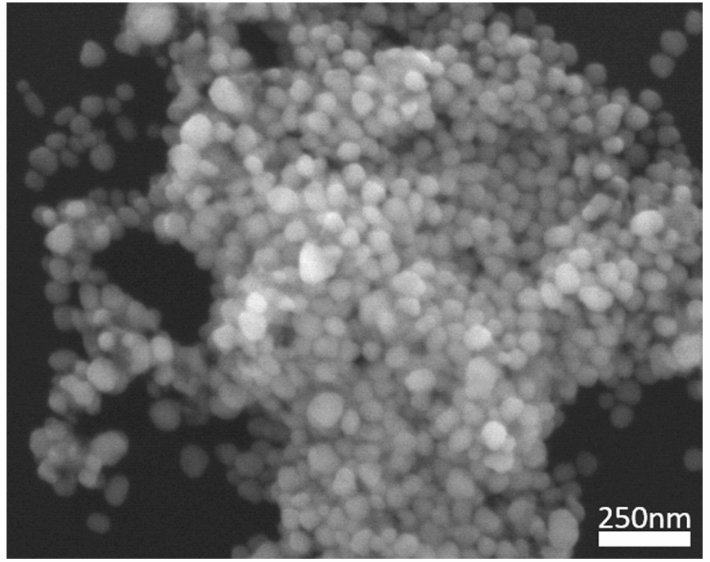


**Figure S4.** The SEM image of as- prepared Ag nanoparticles.


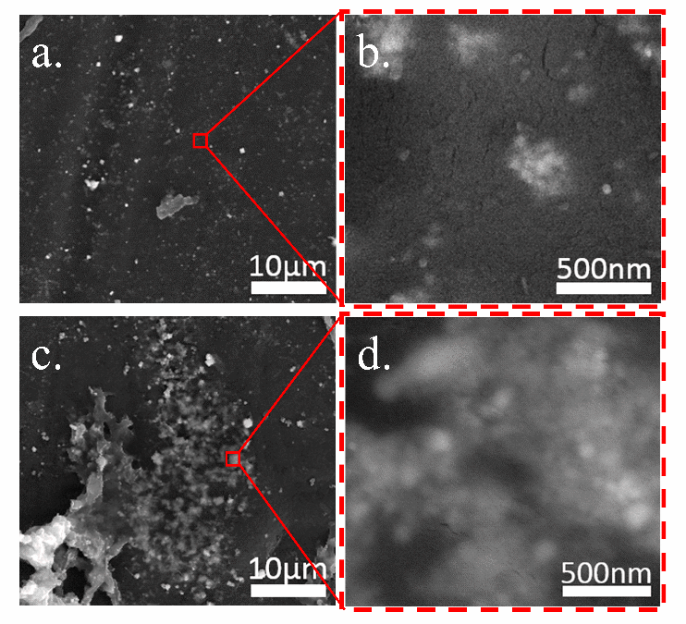


**Figure S5****.** The SEM images of Ag nanoparticles aggregation on the engraved PTFE at the different evaporating temperatures with different multiples. (a)-(b) the evaporating temperature is 20 ^⁰^C; (c)-(d) the evaporating temperature is 70^⁰^C. The whole red small boxes represent the magnified area.

With the increasing concentration of Ag nanoparticles, the Raman intensity dramatically rises and then tends to be stable. With the increase of Ag nanoparticles, there are more “hot spots” on the substrate, leading to the increase of Raman signals. However, when the concentration of Ag nanoparticles reached to 1.19×10^-12^ M, there is not a sharp increase in the Raman intensity. One possible explanation is that Ag nanoparticles would accumulate very thick on the substrate at high concentration. The bottom Ag nanoparticles that are out of the range of the depth of focus contribute relatively weak signals. [[1](#_ENREF_1)] So there is no obvious enhancement of the molecular Raman signals when the Ag colloid solution concentration increases again.To avoid wasting the Ag nanoparticles, the 1.19×10^-12^ M Ag colloid solution is chosen as the initial concentration of Ag nanoparticles.


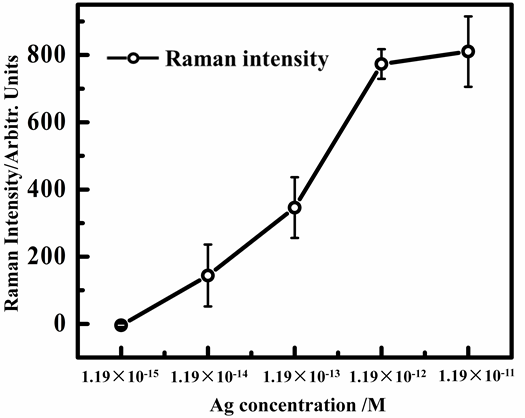


**Figure S6.** The relation between the Ag concentration and the Raman intensity.


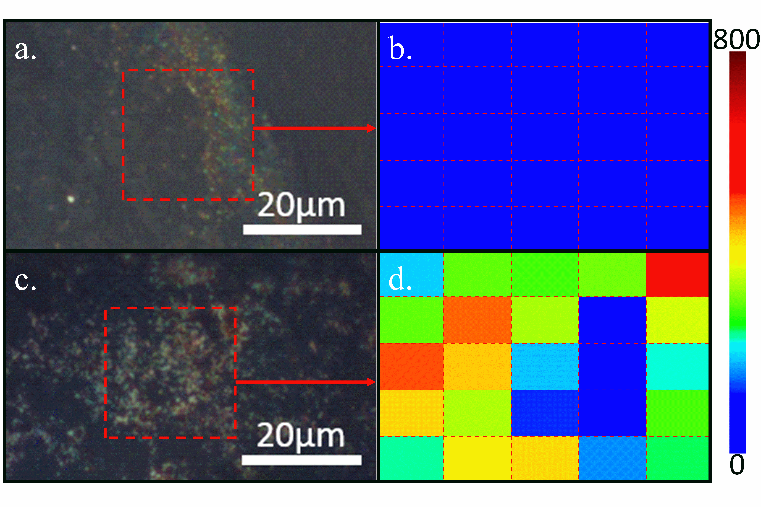


**Figure S7.** SERS mapping results of methylthionine chloride at 1322cm^-1^ obtained from 1× 10^-9^M aqueous solutions on the original PTFE (b)and the engraved PTFE(d). The measurement areas are circled by the homologous red square frame in the (a) and (c).

| Analyte | The limit detection | Detect method/Substrate | Reference |
| --- | --- | --- | --- |
| MB | 1×10^-10^M | Large-scale Ag nanoislands stabilized by a magnetron-sputtered polytetrafluoroethylene film | [[2](#_ENREF_2)] |
|  | 1×10^-17^M | Nanostructured organic semiconductor films | [[3](#_ENREF_3)] |
|  | 150×10^-9^M | The surface energy controlled HAR plasmonic nanopillar array | [[4](#_ENREF_4)] |
|  | 1×10^-14^M | The commercial laser engraved Teflon (PTFE) film with engraved microarray | This work |
| R6G | 1×10^-18^M | Slippery liquid infused-porous surface-enhanced Raman scattering platform | [[5](#_ENREF_5)] |
|  | 1×10^-6^M | A droplet-based high-throughput SERS platform on a droplet-guiding-track-engraved superhydrophobic substrate | [[6](#_ENREF_6)] |
|  | 1×10^-10^M | PLLA nanofibrous paper-based plasmonic substrate | [[7](#_ENREF_7)] |
|  | 1×10^-14^M | The commercial laser engraved Teflon (PTFE) film with engraved microarray | This work |
| BSA | 1×10^-18^M | Slippery liquid infused-porous surface-enhanced Raman scattering platform | [[5](#_ENREF_5)] |
|  | 20 μg/mL | Colorimetric analysis | [[8](#_ENREF_8)] |
|  | 1×10^-4^M | Lithographed gold nanocylinders obtained by the EBL technique | [[9](#_ENREF_9)] |
|  | 0.002 μg/mL | The commercial laser engraved Teflon (PTFE) film with engraved microarray | This work |

**Table S1:** The limit detection of MB, R6G and BSA detected on various substrates or using different methods

**References**

1. S. Y. Chou, C. C. Yu, Y. T. Yen, K. T. Lin, H. L. Chen, and W. F. Su,(2015) "Romantic Story or Raman Scattering? Rose Petals as Ecofriendly, Low-Cost Substrates for Ultrasensitive Surface-Enhanced Raman Scattering," Analytical Chemistry **87**, 6017-6024.

2. M. Šubr, M. Petr, O. Kylián, J. Kratochvíl, and M. Procházka,(2015) "Large-scale Ag nanoislands stabilized by a magnetron-sputtered polytetrafluoroethylene film as substrates for highly sensitive and reproducible surface-enhanced Raman scattering (SERS)," Journal of Materials Chemistry C **3**, 11478-11485.

3. M. Yilmaz, E. Babur, M. Ozdemir, R. L. Gieseking, Y. Dede, U. Tamer, G. C. Schatz, A. Facchetti, H. Usta, and G. Demirel,(2017) "Nanostructured organic semiconductor films for molecular detection with surface-enhanced Raman spectroscopy," Nature materials **16**, 918.

4. S. G. Park, C. Mun, X. Xiao, A. Braun, S. Kim, V. Giannini, S. A. Maier, and D. H. Kim,(2017) "Surface Energy‐Controlled SERS Substrates for Molecular Concentration at Plasmonic Nanogaps," Advanced Functional Materials **27**, 1703376.

5. S. Yang, X. Dai, B. B. Stogin, and T. S. Wong,(2015) "Ultrasensitive surface-enhanced Raman scattering detection in common fluids," Proceedings of the National Academy of Sciences of the United States of America **113**, 268.

6. S. Shin, J. Lee, S. Lee, H. Kim, J. Seo, D. Kim, J. Hong, S. Lee, and T. Lee,(2016) "A Droplet-Based High-Throughput SERS Platform on a Droplet-Guiding-Track-Engraved Superhydrophobic Substrate," Small **13**, 1602865.

7. J. Shao, L. Tong, S. Tang, Z. Guo, H. Zhang, P. Li, H. Wang, C. Du, and X. Yu,(2015) "PLLA nanofibrous paper-based plasmonic substrate with tailored hydrophilicity for focusing SERS detection," Acs Applied Materials & Interfaces **7**, 5391.

8. S. Yan, Y. Zhu, S. Y. Tang, Y. Li, Q. Zhao, D. Yuan, G. Yun, J. Zhang, S. Zhang, and W. Li,(2018) "A rapid, maskless 3D prototyping for fabrication of capillary circuits: Toward urinary protein detection," Electrophoresis **39**, 957-964.

9. C. David, N. Guillot, H. Shen, T. Toury, and D. L. C. Ml,(2011) "SERS detection of biomolecules using lithographed nanoparticles towards a reproducible SERS biosensor," Nanotechnology **21**, 475501-475501.
